# Associations between symptoms of maternal postpartum depression, gestational age and infant social withdrawal: A longitudinal study in a community cohort

**DOI:** 10.1111/bjdp.12414

**Published:** 2022-04-29

**Authors:** Anne Christine Stuart, Maria Stougård, Johanne Smith‐Nielsen, Ida Egmose, Antoine Guedeney, Mette Skovgaard Væver

**Affiliations:** ^1^ Department of Psychology University of Copenhagen Copenhagen Denmark; ^2^ Université de Paris et Hôpital Bichat Claude Bernard APHP Paris France

**Keywords:** gestational age, infant social withdrawal, maternal postpartum depression, partner postpartum depression

## Abstract

Infant social withdrawal is a risk factor for non‐optimal child development; thus, it is important to identify risk factors associated with withdrawal. In a large community sample (*N* = 19,017), we investigate whether symptoms of maternal and partner postpartum depression (PPD; measured with the Edinburgh Postnatal Depression Scale) and prematurity are predictors of infant social withdrawal (measured with the Alarm Distress Baby Scale). Withdrawal was assessed at 2–3, 4–7 and 8–12 months postpartum. Linear regressions showed that prematurity predicted higher infant social withdrawal at all time points, and maternal symptoms of PPD were positively associated with withdrawal at 2–3 months. Logistic regressions showed that odds for elevated social withdrawal were increased with elevated levels of maternal symptoms of PPD at 2–3 and 8–12 months. Partner's symptoms of PPD were not associated with withdrawal. Future studies should investigate how PPD symptoms and prematurity may impact the individual development of social withdrawal.

**Statement of contribution**

***What is already known about this topic***

- Infant social withdrawal is a risk factor for non‐optimal child development.
- Maternal and partner postpartum depression and gestational age have been associated with infant social withdrawal.

***What this paper adds***

- Symptoms of maternal postpartum depression predict infant social withdrawal at 2–3 and 8–12 months.
- Prematurity predicts infant social withdrawal at 2–3, 4–7 and 8–12 months.

## INTRODUCTION

Infants engage in social interactions with their parents from birth. Social withdrawal of shorter duration is a natural part of this interaction as part of the infant's normal regulatory behaviour (Braarud et al., 2013; Guedeney, 1997). When the infant withdraws from the social interaction, it helps regulate the flow of stimulation and indicates that the infant needs a break in the parent–child interaction (Tronick, 2007). However, if these acts of withdrawal are prolonged and characteristic of a social interaction, it may be an early alarm signal of serious infant distress (Ulak et al., 2020; Zeanah et al., 2000). Studies have found that infant social withdrawal is associated with neurodevelopmental and psychological disorders, for example, autism spectrum disorders, anxiety disorders (Egger & Angold, 2006; Viaux‐Savelon et al., 2010) and a key predictor of infant depression (Guedeney, 2007). Further, withdrawal is linked with insecure (Rubin & Lollis, 1988) and disorganized attachment (Solomon & George, 1999). Infant social withdrawal has been associated with poorer cognitive and language development (Milne et al., 2009) and emotional and behavioural problems at 3–5 years (Guedeney et al., 2014). Thus, infant social withdrawal may be a risk factor for non‐optimal child development, whereby it is important to identify and understand the possible risk factors for prolonged infant social withdrawal.

It is generally acknowledged that infants born preterm may be at risk for developing major impairments, as the risk increases as gestational age decreases (Allen, 2008). Prematurity has been both associated with biological impairments (Escobar et al., 2006; Gouyon et al., 2012) as well as a social delay (Guedeney et al., 2012). Previous studies have found an association between prematurity and increased levels of social withdrawal in infancy. Braarud et al. (2013) compared infants born prematurely, that is, week 30–36, (*n* = 64), to full‐term (*n* = 238) at 3, 6 and 9 months postpartum. Social withdrawal was measured using the Alarm Distress Baby Scale (ADBB; Guedeney & Fermainen, 2001). The ADBB is a short observer‐rated screening instrument used to detect social withdrawal in infants 2–24 months. Results showed premature infants had higher social withdrawal scores at 3 and 6 months but not at 9 months. Braarud and colleagues argue that the group differences are most profound in the first 6 months of the infant's life, and they suggest this may be due to the mother's higher educational level and longer parental leave in Norway act as protective factors in the long run. Another study using the same sample found a negative correlation between social withdrawal and gestational age as well as with birth weight (Moe et al., 2016), indicating these as risk factors for infant social withdrawal. Accordingly, a large study of 12**‐**month**‐**old infants (*N* = 1586) by Guedeney et al. (2012) found the probability for an infant being socially withdrawn (as measured with the ADBB) was higher for premature infants compared with full‐term. They argue that particularly in premature infants, social withdrawal behaviour is an important alarm signal to detect early on.

Another factor associated with infant social withdrawal is maternal mental health, particularly postpartum depression (PPD) and symptoms thereof. Maternal PPD has long been known to negatively affect parent–child interactions (Field et al., 1990) and infant socioemotional and cognitive development (Murray et al., 2010). Infants of mothers with PPD are found to show more negative affect and be less engaged when interacting with the mother (Dollberg et al., 2006; Feldman et al., 2009; Tronick & Reck, 2009). Further, infants of mothers with PPD are found to engage in more self‐regulatory behaviour (Tronick, 2007), which resembles infant social withdrawal behaviour (Guedeney, 2007). This possible link between maternal PPD and infant social withdrawal has been explained by the fact that depression impacts the mother's ability to function as an adequate partner for the infant to learn social behaviour as mothers suffering from PPD are shown to be less sensitively attuned to their infant's needs (Field, 2010; Væver et al., 2020). The infants, therefore, withdraw socially from the interaction in order to cope with the suboptimal parenting (Beebe et al., 2008; Feldman et al., 2009). This possible link between maternal PPD and infant social withdrawal has been investigated in a number of empirical studies. In the aforementioned study by Braarud et al. (2013), level of maternal depressive symptoms was measured with the Edinburgh Postnatal Depression Scale (EPDS; Cox et al., 1987). For the full‐term infants, social withdrawal assessed at 9 months correlated positively with PPD symptoms measured at 3, 6 and 9 months postpartum. Surprisingly for the premature infants, social withdrawal at 9 months correlated negatively with PPD symptoms at 6 months. The authors argue that the higher EPDS scores may reflect mothers worrying about medical risks of the prematurely born infant rather than maternal mood. Thus, the mothers may have stimulated their child more, resulting in the lower social withdrawal scores at 9 months, and Braarud et al. argue that it may take longer to detect the effect of maternal PPD symptoms on social withdrawal in premature infants. However, despite being significant, all of the correlations were relatively small (*r*s = .14–.28). Two recent studies also found that maternal PPD symptoms scores predicted infant social withdrawal, with higher levels of depressive symptoms being associated with higher scores of social withdrawal (Puura et al., 2019; Smith‐Nielsen et al., 2019). Puura and colleagues investigated a community sample of 113 mother–infant dyads at 7 months postpartum and measured PPD symptoms with the EPDS and infant social withdrawal with the ADBB. The Smith‐Nielsen et al. study assessed 28 dyads with the mother having PPD and 41 control dyads at 4 months. PPD symptoms were assessed with the EPDS and infant social withdrawal with the ADBB. One study by Matthey et al. (2005) also assessed 44 infants at 13–52 weeks at a routine physical check‐up, using the ADBB and EPDS as measures for social withdrawal and level of depressive symptoms, respectively. They found no significant association between the EPDS and infant social withdrawal but that a single questionnaire item of the mother's mood since birth did significantly predict the ADBB score at 7 months. Thus, the authors argue that infant social withdrawal was only predicted by mother's retrospective reports of PPD and not by concurrent symptoms. Finally, a study by Dollberg et al. (2006) investigated the relationship between maternal PPD (measured with the Beck Depression Inventory, BDI; Beck, 1978) and infant social withdrawal (measured with the ADBB) in 36 clinic‐referred and 43 control infants at 7–36 months. They found no association between maternal PPD and infant social withdrawal. Instead, they found an association between depressed maternal behaviour, that is, sad affect, reduced energy, apathy and gaze aversion and social withdrawal during feeding and play.

While evidence emerges of maternal PPD symptoms as a risk factor for social withdrawal, to our knowledge, only one study has examined the influence of father's level of depressive symptoms. Mäntymaa et al. (2008) investigated 260 infants from a population‐based sample aged 4, 8 and 18 months, with 10 infants in total considered socially withdrawn as measured with the ADBB. Both mothers' and fathers' levels of depressive symptoms were assessed using the EPDS. Further, they also developed their own questionnaire for both parents on their perceived mental health. They found that mothers with high levels of depressive symptoms were more likely to have socially withdrawn infants, but father's level of depressive symptoms was not. However, the probability for the infant being socially withdrawn was higher if both parents reported either more symptoms of depression or poor perceived mental health compared with families where only one parent reported high levels of symptoms and the other parent reported good mental health/low levels of depressive symptoms. This may indicate that the partner's PPD symptoms in itself are not a predictor of infant social withdrawal, but the combination of either one or both parents having high levels of depressive symptoms is a potential risk factor for the infant.

The present longitudinal study investigated in a large community sample the relationship between mother's and partner's levels of depressive symptoms, gestational age and infant social withdrawal. We test the following hypotheses that: (1) higher levels of maternal depressive symptoms predict higher levels of infant social withdrawal at 2–3 months, 4–7 months and 8–12 months postpartum; (2) premature infants show higher levels of social withdrawal compared with full‐term infants at the three time points; and (3) prematurity moderates the relationship between maternal depressive symptoms and infant social withdrawal at the three time points, that is, the relationship between maternal PPD and infant social withdrawal is stronger among premature infants. We also expect (4) elevated maternal depressive symptoms (indicated by mothers scoring above cut‐off on the EPDS) will predict elevated infant social withdrawal (indicated by infants scoring above cut‐off) at the three time points. Finally, we expect (5) partner's level of depressive symptoms moderates the relationship between maternal depressive symptoms and infant social withdrawal at the three time points, that is, high levels of depressive symptoms in both mother and partner lead to higher infant social withdrawal.

## METHODS

### Participants and procedure

**FIGURE 1:**
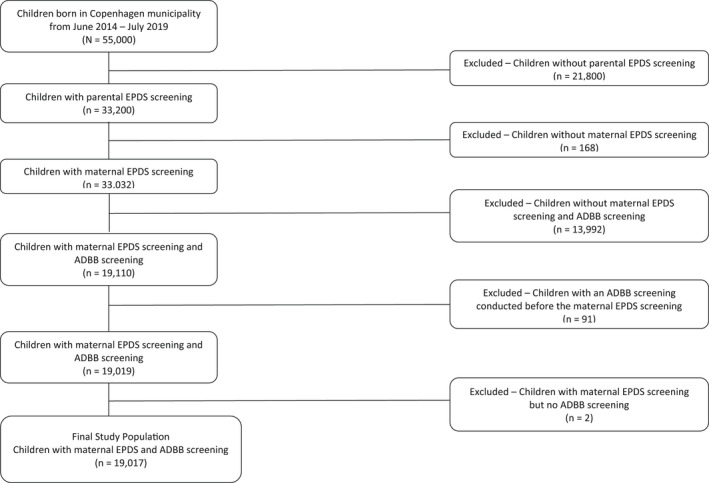
Flow chart of the current study sample

The study protocol for the current study was pre‐registered at osf.io/vqe29. The cohort consisted of all infants aged 2–12 months born in the municipality of Copenhagen between 1 July 2014 and 15 July 2019 and their mothers and partners. All families in Copenhagen with a newborn child can accept to receive routine home visits by a public health visitor as part of the national social security and health system. The specific numbers of acceptances are unknown, but in 2017, 96.9% of families were visited at 2 months old in 33 municipalities (Pedersen et al., 2017). The health visitors are automatically informed about all deliveries and visit the families at least four times during the first year after the birth. Visits occur when the infant is approximately 1–14 days, 2–3, 4–7 (only first‐time families) and 8–10 months old. Each visit includes anthropometric measuring, evaluation of motor and speech development, guidance of infants' emotional and developmental needs and feeding, supporting the parents in their new roles, and screening for infant social withdrawal and maternal depression with the ADBB and the EPDS, respectively. All visits are reported in the electronic health visitor journals that also include birth information of the infants. Data were extracted from the electronic Danish Health Visitor Journals. Inclusion criteria were as follows: infants aged 2–12 months born in the municipality of Copenhagen during the study period with minimum one ADBB screening, and mothers had minimum one EPDS screening. Exclusion criteria were as follows: invalid personal identification number, stillborn (the infant's death date is the same as their birth date), infants with a death date before their birth date, infants born outside of the study period, incomplete ADBB and EPDS screenings and ADBB screenings prior to mother's registered EPDS screening were excluded (if they took place on the same date, they were included). The final sample consisted of 19,017 mother–infant dyads (see Figure 1 for flow chart). The partner's EPDS score was available for about 18% of this sample (i.e. 3557). Table 1 shows the sample characteristics. Gestational age was categorized into full‐term (37–46 weeks) and premature (30–36 weeks). We did not have information on gestational age for 321 infants (1.7%).

**TABLE 1:** Sample characteristics

Maternal age at birth (years)	
*M*	32.0
*SD*	4.6
Partner's age at birth (years)	
*M*	33.8
*SD*	4.8
Gestational age	
Premature, *n* (%)	888 (4.7%)
Full‐term, *n* (%)	17,808 (93.6%)
Birth weight (kg)	
*M*	3.49
*SD*	0.51
Child sex	
Boy, *n* (%)	9667 (50.8%)
Girl, *n* (%)	9350 (49.2%)

*Note*: Premature = 30–36 weeks, full‐term = 37–46 weeks.

### Measures

#### Edinburgh postnatal depression scale

The EPDS is used to screen for symptoms of PPD occurring in the past 7 days for mother and partners. It is a self‐report questionnaire consisting of 10 items relating to mood and feelings (Cox et al., 1987). The items are rated on a 4‐point Likert scale. Total scores range from 0 to 30 points with higher scores indicating more severe symptoms. In a Danish setting, a cut‐off score of 11 has been identified as optimal for depression according to both DSM‐5 and ICD‐10 (Smith‐Nielsen et al., 2018). This corresponds to findings from a recent meta‐analysis (Levis et al., 2020). Mother's and partner's first registered EPDS scores were used in the analyses.

#### Alarm distress baby scale

The ADBB is used to assess infant social withdrawal (Guedeney & Fermanian, 2001). It is an observational instrument, consisting of eight items rated 0–4, measuring the infant's social behaviour in relation to a stranger (i.e. the health visitor). The eight items are facial expression, eye contact, general level of anxiety, self‐stimulating gestures, vocalizations, response to stimulation, relationship and attraction. Low ADBB scores indicate optimal social behaviour with a cut‐off score of 5 or more being recommended. The health visitors conducted and scored the ADBB assessment at their regular home visits during the first year. The assessment can be done in any social interaction context between the observer (i.e. health visitor) and infant (e.g. paediatric examination, developmental testing or simple face‐to‐face interaction). In this study, ADBB was measured in three waves throughout the infant's first year: wave 1 between 2 and 3 months, wave 2 between 4 and 7 months (only for primiparae) and wave 3 between 8 and 12 months. The first ADBB score in each wave was used in the analyses (a screening is excluded if there are <30 days between them).

### Statistical analyses

All analyses were performed using SPSS Statistics 26 (IBM). To test hypotheses 1–3 and 5, multiple linear moderation analyses were used. ADBB scores at the three different waves were the dependent variable in the different models. Gestational age, mother's EPDS score and an interaction effect between them were the independent variables in all of the models for hypotheses 1–3. EPDS score was mean centred. For hypothesis 5, the independent variables were partner's and mother's EPDS scores and the interaction term between them. EPDS scores were mean centred. As the residuals in the linear regression analyses were not normally distributed, a bias‐corrected and accelerated (BCa) bootstrap procedure was used, and 95% confidence intervals (CI) were used to evaluate these associations (if zero, that is, the null hypothesis, was not included in the interval, it was interpreted as significant). To test hypothesis 4, multiple binary logistic regression analyses were used. Dichotomous ADBB scores (below/above 5) at the three waves were the dependent variable in the separate models. The dichotomous maternal EPDS score (below/above 11) was the independent variable in all of the models. For all analyses, child sex and maternal age at birth were included as covariates as a review found that these were linked to infant social withdrawal (Guedeney et al., 2013). For hypothesis 5, when including partner's EPDS score, partner's age at birth was also included as a covariate. When including partners (who we only had a subset of EPDS data on), we carried out complete case analysis. All reported *p*‐values are two‐tailed and evaluated at a significance level of .05.

## RESULTS

### Descriptive statistics

Table 2 shows descriptive statistics for ADBB scores at the three time waves and mothers' and partners' first EPDS scores. ADBB mean scores were generally low across the three waves. Further, both mothers and partners score low on the EPDS on average, indicating that this is a low‐risk sample.

**TABLE 2:** Descriptive statistics

	Premature		Full‐term		Total	
	*M*	*SD*	*M*	*SD*	*M*	*SD*
Infant ADBB score						
Wave 1 (*n* = 11,323)	1.27	1.57	0.56	1.19	0.59	1.22
Wave 2 (*n* = 8999)	0.53	1.04	0.42	0.98	0.43	0.98
Wave 3 (*n* = 9831)	0.48	1.24	0.31	0.86	0.31	0.89
Mother's EPDS score	5.48	4.15	4.83	3.81	4.86	3.82
Partner's EPDS score	4.43	3.65	3.61	3.13	3.65	3.17

*Note*: Premature = 30–36 weeks, full‐term = 37–46 weeks. The same infant can be in more than one wave.

Abbreviations: ADBB, alarm distress baby scale; EPDS, Edinburgh postnatal depression scale.

Table 3 shows how many infants scored above/below cut‐off (≥5) on the ADBB at the three waves and how many mothers scored above/below the cut‐off (≥11) EPDS. As shown, the vast majority of the sample scored below cut‐off.

**TABLE 3:** Frequencies of infants and mothers scoring above/below the recommended cut‐offs on the ADBB and EPDS measures

	Above cut‐off		Below cut‐off	
	*n*	%	*n*	%
Infant ADBB				
Wave 1	140	1.2	11,336	98.8
Wave 2	55	0.6	9115	99.4
Wave 3	52	0.5	9963	99.5
Maternal EPDS	1524	8.0	17,493	92.0

Abbreviations: ADBB, Alarm Distress Baby Scale, recommended cut‐off ≥5; EPDS, Edinburgh Postnatal Depression Scale, recommended cut‐off ≥11.

### Associations between gestational age, mother's EPDS and ADBB scores

Linear moderation analyses showed that at wave 1 (i.e. 2–3 months), gestational age was a significant predictor (*b* = −0.68, SE = 0.07, *p* = .001, 95% CI [−0.82; −0.56], *r*_sp_ = −.12), meaning that infants born preterm had a significantly higher ADBB score compared with full‐term. Maternal EPDS score was also a significant predictor (*b* = 0.05, SE = 0.02, *p* = .005, 95% CI [0.02; 0.09], *r*_sp_ = .04), reflecting that the higher the mother scored on the EPDS, the higher the ADBB score was. There was no significant moderation effect (*b* = −0.02, SE = 0.02, *p* = .418, 95% CI [−0.05; 0.02], *r*_sp_ = −.01). When the infant was 4–7 months, that is, wave 2, gestational age was still a significant predictor (*b* = −0.10, SE = 0.05, *p* = .031, 95% CI [−0.20; −0.02], *r*_sp_ = −.02), but maternal EPDS score was not (*b* = 0.01, SE = 0.01, *p* = .430, 95% CI [−0.01; 0.03], *r*_sp_ = .01), and there was no significant moderation effect (*b* = 0.004, SE = 0.01, *p* = .729, 95% CI [−0.02; 0.02], *r*_sp_ = .00). At wave 3, that is, 8–12 months, gestational age was still a significant predictor (*b* = −0.17, SE = 0.06, *p* = .005, 95% CI [−0.32; −0.04], *r*_sp_ = −.04), but there was no significant main effect of mother's EPDS score (*b* = 0.004, SE = 0.02, *p* = .784, 95% CI [−0.02; 0.03], *r*_sp_ = .00) or moderation effect (*b* = 0.005, SE = 0.02, *p* = .743, 95% CI [−0.03; 0.04], *r*_sp_ = .01).

### Associations when using the mother's EPDS and ADBB scores categorically

Binary logistic regression analyses showed that if mothers scored above 11 on the EPDS, odds increased by 4.47 for the infant to score ≥5 on the ADBB at wave 1 (Wald χ^2^(1) = 60.98, *p* < .001, 95% CI [3.07; 6.51]). At wave 2, there was no significant association between maternal EPDS and ADBB (OR = 1.55, Wald χ^2^(1) = 1.15, *p* = .284, 95% CI [0.70; 3.43]). At wave 3, odds for infant scoring ≥5 on the ADBB increased by 2.95 if their mother scored ≥11 compared with mothers who score below the cut‐off on the EPDS (Wald χ^2^(1) = 9.36, *p* = .002, 95% CI [1.48; 5.91]).

### The effect of partner's EPDS scores on maternal EPDS and infant ADBB scores

Linear moderation analyses showed that maternal EPDS score was a significant predictor of ADBB scores at wave 1 (*b* = 0.04, SE = 0.01, *p* = .001, 95% CI [0.02; 0.06], *r*_sp_ = .12). However, partner EPDS score did not significantly predict ADBB score (*b* = 0.004, SE = 0.01, *p* = .687, 95% CI [−0.02; 0.03], *r*_sp_ = .01), and there was no significant interaction effect between the two (*b* = −0.002, SE = 0.002, *p* = .260, 95% CI [−0.00; 0.01], *r*_sp_ = .03). At wave 2 and wave 3, there were no significant main effects (all *p*s ≥ .08, 95% CI [−0.00; 0.02], all *r*_sp_s ≤ .04) or moderation effects (all *p*s ≥ .064, 95% CI [0.00; 0.01], all *r*_sp_s ≤ .04).

## DISCUSSION

The present study investigated maternal PPD symptoms, gestational age and partner PPD symptoms as possible predictors of infant social withdrawal as measured with the ADBB at 2–3, 4–7 and 8–12 months postpartum.

Our first hypothesis was partly supported as we found that maternal PPD symptoms significantly predicted infant social withdrawal at 2–3 but not at 4–7 or 8–12 months. Previous studies have also found a significant association between maternal level of depressive symptoms and infant social withdrawal in the same age group, measured with the EPDS and ADBB, making our results comparable (Braarud et al., 2013; Puura et al., 2019; Smith‐Nielsen et al., 2019). However, two studies did not find a significant association, with one study using BDI instead of EPDS and assessing 7–36‐month‐old children though the ADBB is only validated for up to 24‐month‐old infants, as well as measuring withdrawal with the mother instead of with a stranger (Dollberg et al., 2006). However, while the association between the measures was not significant, they did find a significant association between specific maternal behaviours characteristic of depression and infant social withdrawal. Matthey et al. (2005) also did not find a significant association between the EPDS and ADBB, but maternal retrospective report of mental health and PPD was associated with infant social withdrawal. Thus, it seems fairly well established that maternal PPD symptoms are associated with infant social withdrawal in the first year postpartum, both when assessed concurrently and when maternal PPD is used as a predictor. One possible reason that we did not find a significant effect at 4–7 months may be due to it only being first‐time mothers who receive a visit from the health visitor at this time point. Only one of the previous studies solely investigated primiparous mothers (Smith‐Nielsen et al., 2019), finding a significant association between EPDS and ADBB score at 4 months. While it is somewhat surprising we did not find an effect in this age group, the difference may be due to the age range in our study (4–7 months) compared with infants all measured at 4 months. Future research should investigate the relationship between maternal PPD symptoms and infant social withdrawal in first‐time mothers further. However, we also did not find a significant association at 8–12 months. Considering previous studies, this was a surprising finding. Braarud et al. (2013) found that infant social withdrawal at 9 months correlated positively with EPDS at 3, 6 and 9 months. Contrary, our findings may indicate that symptoms of PPD measured in the first months of the infant's life may not predict infant social withdrawal later at 4–7 and 8–12 months. It is of note, however, that in our study, the vast majority of EPDS and ADBB scores at 2–3 months were measured on the same date (84.1% of all cases), meaning that we are unable to establish a causal relationship. Thus, it may be that maternal symptoms of PPD only affect infant social withdrawal concurrently and not longitudinally. In the present study, we included the mother's first EPDS score and thus do not know if the level of symptoms varies across the first year of the infant's life.

The second hypothesis that premature infants scored higher on social withdrawal compared with full‐term was confirmed at all three time points. Previous studies have all found prematurity associated with higher scores of infant social withdrawal during the infant's first year of life (Braarud et al., 2013; Guedeney et al., 2012; Moe et al., 2016). It was only at 9 months Braarud and colleagues did not find a significant association; however, they did not explain why. Our results demonstrate that premature infants are at an increased risk for infant social withdrawal, putting them at risk for later developmental disorders and psychopathology (for review, see Hayes & Sharif, 2009).

Contrary to our third hypothesis, none of the moderation effects of maternal PPD symptoms and gestational age were significant. To our knowledge, no previous studies have investigated how maternal PPD symptoms and gestational age may interact and affect infant social withdrawal scores, but Braarud et al. (2013) did investigate how EPDS and ADBB correlated independently in the two groups. They found that the two measures correlated significantly at all three time points in the full‐term group, but for the premature infants, they only correlated at one time point. However, when conducting separate correlation analyses, the authors did not control for group status, which may have biased the results. Our findings indicate that maternal PPD symptoms and gestational age do not interact and should thus be seen as independent risk factors that do not accumulate in regard to affecting infant social withdrawal. However, these non‐significant results may also be due to sample characteristics. This sample is a community sample with a relatively low EPDS mean score relative to the cut‐off score of 11. Maternal PPD symptoms are assessed as a self‐reported level of depressive symptoms and not as a diagnosis. If investigated in a clinical sample, it is likely the combination of high levels of PPD symptoms and low gestational age increases the risk of infant social withdrawal. This would be in line with the cumulative risk hypothesis (Rutter, 1979), which posits that as the *number* of risk factors increases (i.e. clinical PPD diagnosis and prematurity), the likelihood of negative outcomes also increases (i.e. higher level of infant social withdrawal). Future studies should investigate whether maternal PPD symptoms and gestational age interact and increase the risk of infant social withdrawal in different at‐risk populations. However, it is also important to consider that there may be other risk factors explaining infant social withdrawal, either as main effects or interacting with symptoms of PPD or gestational age. For example, studies have found that factors such as other mental health disorders (Carter et al., 2001; Crugnola et al., 2016) or parental conflict (Chen et al., 2015) mediate the adverse effects of PPD on the child's mental health and behaviour. Future studies should, therefore, also study other risk factors apart from PPD and gestational age in relation to infant social withdrawal.

Our fourth hypothesis was partly supported as we found that elevated maternal depressive symptoms predicted infant social withdrawal (i.e. a score above the cut‐off ≥5) at 2–3 and 8–12 months but not at 4–7 months. To our knowledge, no previous study has investigated the relationship between EPDS and ADBB when used categorically. However, the results based on categorical data are consistent with the results based on continuous data, indicating they are comparable. The results showed that the odds for scoring above cut‐off on the ADBB increased for infants of mothers scoring above cut‐off on the EPDS (score ≥ 11) compared with infants of mothers with non‐elevated levels of depressive symptoms. All of our results seem to indicate that level of maternal depressive symptoms – measured both continuously and categorically – is a significant risk factor of infant social withdrawal. When measured categorically, we found an effect of maternal depressive symptoms (measured around 2 months) on infant social withdrawal measured at 8–12 months that was not found when using the measures continuously. This may indicate a threshold effect, meaning only infants of mothers scoring above cut‐off, that is, mothers who are likely to have clinical levels of depression during the first year postpartum, are likely to show social withdrawal both concurrently and longitudinally. However, one important factor to consider with these categorical results is the low percentage of the sample scoring above cut‐offs on the ADBB and EPDS as would be expected in the general population. The very skewed group sizes can influence the results and should, therefore, be interpreted with caution. Further, the cut‐off of 5 on the ADBB has also not been validated in a Danish context in a low‐risk community sample. It may, therefore, be that an infant scoring 4 or even 3 on the ADBB is showing elevated levels of social withdrawal. This may also explain why there are so few infants scoring above cut‐off in our sample. It is also important to consider whether there are some items that are better at explaining social withdrawal. A recent study in the same sample found that the items self‐stimulating gestures (item 4), vocalizations (item 5) and response to stimulation (item 6) may be less useful when using the ADBB as a universal screening instrument in a community sample (Egmose et al., 2021). Thus, future research should investigate how both a lower cut‐off and a modified version of the ADBB would be more useful as a screening instrument in low‐risk samples.

Finally, we were not able to confirm the fifth hypothesis about partner's level of depressive symptoms as a moderator of the relationship between maternal PPD and infant social withdrawal at any of the three waves. Therefore, in this sample, increased symptoms of depression in the partner did not magnify the effect of maternal PPD on infant social withdrawal. To our knowledge, only one other study has investigated the role of partner PPD symptoms on this relationship, finding the probability of the infant scoring above cut‐off on the ADBB was higher if both parents reported poor mental health (Mäntymaa et al., 2008). The difference in results may be due to different measurements. While both studies used ADBB and EPDS to assess infant social withdrawal and level of depressive symptoms, Mäntymaa and colleagues also included a self‐made questionnaire where the parents reported their perceived mental health. When conducting the analyses, level of depressive symptoms and perceived mental health were combined into one score when investigating the combined effect of both parents reporting mental health problems on infant social withdrawal. In an exploratory analysis, the authors found that fathers' perceived mental health and mothers' depressive symptoms were the factors most strongly contributing to infant social withdrawal. Thus, it may be that paternal level of depressive symptoms **is** not a relevant risk factor for infant social withdrawal. Studies have also found that the EPDS may not be a valid instrument for screening PPD symptoms for fathers (Carlberg et al., 2018; Massoudi et al., 2013), and it may be that Mäntymaa and colleagues' questionnaire is a better measurement of poor mental health in fathers, leading to the difference in results. Another important factor to consider is that we only had the partner's EPDS score on a subset of the entire sample (ca. 18%). It may be that the health visitors only gave the EPDS to specific partners, for example, those who were already interested and prioritized being present at the health visitor's visit. In Denmark in the time period of the present study, fathers were entitled to 14 weeks of paid leave if they wished, and mothers were entitled to the same amount of weeks. A further 32 weeks of parental leave could be shared as they liked, with Danish mothers taking the majority and Danish fathers taking 29–34 days of leave in the time period of 2015–2019 (Danmarks Statistik, 2021). Prentow & Madsen (2017) found around a third of fathers present when the health visitor screened for PPD and ca. 7% of these met the criteria for a PPD diagnosis. Our results may, therefore, be affected due to selection bias, and partner PPD could play a larger role than we are able to detect. Future studies should further investigate the role of partner's level of depressive symptoms as a risk factor in itself as well as combined with maternal level of depressive symptoms on infant social withdrawal.

For all of the significant results reported in the present study, it is important to mention that the effect sizes are small. With larger sample sizes, the statistical power increases, and if the sample size is large enough, a non‐null comparison will always show as statistically significant unless the population effect size is exactly zero (Faber & Fonseca, 2014; Sullivan & Feinn, 2012). Thus, while we have detected statistically significant associations between our study variables, they may not be clinically meaningful. It is, therefore, important to interpret the effect sizes, which are relatively small. Therefore, although maternal PPD symptoms and gestational age are significant predictors of infant social withdrawal at a group level, they can only explain a relatively small amount of the variance in ADBB scores, and hence, other factors may potentially better explain and predict infant social withdrawal. Especially considering this sample could be characterized as low‐risk due to very low EPDS and ADBB scores, it may be that other variables are better at explaining and predicting infant social withdrawal, for example, background variables like socio‐economic status or feeding (for review, see Guedeney et al., 2013). Future studies should further try to identify possible risk factors of infant social withdrawal.

The present study has some limitations. First, we did not have any socio‐economic information on the participants, meaning we were not able to control for, for example, education. Maternal education is a well‐known covariate for affecting mother–child interactions, and it should, therefore, be explored further how controlling for socio‐economic status may influence our results. We know that in the municipality, 78.3% of women aged 19–44 had a middle‐to‐long formal education and were employed (Danmarks Statistik, 2022), meaning that the general population in Copenhagen can be described as a well‐resourced population. Second, and related to the first limitation, the cohort only included data from families in Copenhagen, meaning the results may not be generalizable to the broader population or high‐risk samples. Third, the ADBB and EPDS scores at the first wave may have been measured at the same day, meaning it is not possible to test for a causal relationship between the two at this time point. Fourth, assessing paternal PPD with the EPDS has not been validated in a Danish context; however, Swedish studies have tried to validate the instrument and tentatively concluded it may not be a sufficient screening instrument for PPD in fathers (Carlberg et al., 2018; Massoudi et al., 2013). Future studies should try to validate the EPDS in a Danish context or use another measure for assessing paternal PPD.

Our study also has a number of strengths. First, the sample size is the largest to date, making it possible to do subgroup analyses and analyses with categorical measured data (even though the majority of participants score low on the EPDS and ADBB) without suffering a major power loss. Second, the data were collected by health visitors who visited all families with a newborn, resulting in most mothers and infants being included in the target population, minimizing the risk of selection bias.

In conclusion, the present study finds that both maternal depression and prematurity are risk factors for infant social withdrawal during the first year of life. We were not able to confirm the role of partner depression on social withdrawal, and future studies should investigate further using well‐validated instruments. The present study has identified important predictors of infant social withdrawal, but it has not investigated how infant social withdrawal may change for the individual infant during its first year of life as a function of age and change in parental depressive symptoms. Future studies should investigate how PPD symptoms and gestational age may impact the individual development of social withdrawal.

## CONFLICT OF INTEREST

The authors declare that there is a potential conflict of interest, since the Center for Early Intervention and Family Studies, where five of the authors are employed, offers training in the use of the ADBB scale as part of the continuing education programme at the University of Copenhagen.

## AUTHOR CONTRIBUTIONS

**Maria Stougård:** Conceptualization; Data curation; methodology; Writing–review&editing. **Johanne Smith‐Nielsen:** Conceptualization; methodology; Writing–review&editing. **Mette Skovgaard Væver:** Conceptualization; Funding acquisition; supervision; Writing–review&editing. **Ida Egmose:** Conceptualization; methodology; Writing–review&editing. **Anne Christine Stuart:** Conceptualization; Formal analysis; methodology; Writing–Original draft.

## Data Availability

The data that support the findings of this study are available on request from the corresponding author. The data are not publicly available due to privacy or ethical restrictions.
